# Genome-Wide Identification and KASP Validation of Two Host Genetic Loci Associated with Bovine Leukemia Virus Proviral Load

**DOI:** 10.3390/pathogens15101017

**Published:** 2026-09-26

**Authors:** Yingbao Ye, Sonoko Watanuki, Fumihiro Nagata, Yasunobu Matsumoto, Yoshiyuki Miyazaki, Yoko Aida

**Affiliations:** 1 Laboratory of Global Infectious Diseases Control Science, Graduate School of Agricultural and Life Sciences, The University of Tokyo, 1-1-1 Yayoi, Bunkyo-ku, Tokyo 113-8657, Japan; 2 Laboratory of Global Animal Resource Science, Graduate School of Agricultural and Life Sciences, The University of Tokyo, 1-1-1 Yayoi, Bunkyo-ku, Tokyo 113-8657, Japan; 3 Livestock Improvement Association of Japan, 312 Kanamaru-machi, Maebashi 371-0121, Gunma, Japan; y-miyazaki@liaj.or.jp

**Keywords:** bovine leukemia virus, proviral load, genome-wide association study, single-nucleotide polymorphisms, BoLA, KASP, genetic biomarker

## Abstract

The proviral load (PVL) of bovine leukemia virus (BLV) is associated with viral transmission and disease progression to BLV-induced enzootic bovine leukosis and is influenced by genetic polymorphisms of the bovine leukocyte antigen (*BoLA*)-*DRB3* gene. However, its host genetic determinants remain partially defined because additional host events are necessary for BLV infection outcomes. We performed a genome-wide association study of 113 BLV-positive Holstein cattle to identify novel single-nucleotide polymorphisms (SNPs). We first compared 33 high-PVL and 37 low-PVL cattle using 582,324 quality-controlled SNPs. Two SNPs, rs110742604 and rs137368875, exceeding the Bonferroni-corrected significance threshold, were identified in the *BoLA* class I region of chromosome 23. Kompetitive allele-specific PCR (KASP) assays were developed for both SNPs, and three genotype calls (TT, TC, and CC) were concordant with Sanger sequencing in all samples. Further, genotyping was performed for rs110742604 and rs137368875 in 364 and 362 cattle, respectively. At both loci, the C allele was associated with a lower PVL, with the lowest PVL observed in CC animals. In a combined genotype of three resistance markers, *BoLA-DRB3* resistance-associated alleles, rs110742604-CC and rs137368875-CC, stepwise enrichment in cattle with low PVL was dependent on the increasing number of candidate resistance markers; further, 11 of 12 cattle carrying all three markers showed a tendency toward low PVL, and none had high PVL. These findings identify candidate host biomarkers for BLV PVL and establish rapid assays for large-scale validation. Replication across breeds and functional characterization are required before incorporation into genomic selection or integrated control programs for BLV.

## 1. Introduction

Bovine leukemia virus (BLV), belonging to the *Deltaretrovirus* genus of the *Retroviridae* family, establishes lifelong infection and causes enzootic bovine leukosis (EBL). Most infected cattle remain clinically asymptomatic; approximately one-third develop persistent lymphocytosis, and only a small proportion progress to B-cell lymphoma after a prolonged latency period [1,2,3]. Despite the relatively low incidence of overt malignancy, BLV remains important because infected animals constitute a long-term reservoir, and infection is widespread in major cattle-producing regions [4]. A nationwide survey conducted between 2009 and 2011 in Japan found BLV seropositivity in 40.9% of dairy cattle and 28.7% of beef breeding cattle [5]. In countries or herds with a high prevalence, test-and-cull programs can be economically difficult to sustain, indicating the need for control strategies that prioritize animals based on their contribution to transmission and disease risk. The impact of BLV is not limited to clinical lymphoma: infection has been associated with reduced carcass value, shortened productive life, and lower milk production [6,7,8,9,10,11]. Chronic BLV infection can also impair immune function, potentially increase susceptibility to secondary diseases, and amplify veterinary and management costs [12,13,14].

As a retrovirus, BLV integrates its genome into host DNA as a provirus, resulting in lifelong infection. The BLV proviral load (PVL), defined as the number of integrated proviral copies, is a central quantitative marker of infection status. The BLV-CoCoMo-qPCR system enables sensitive quantification across diverse BLV variants [15], and subsequent studies have demonstrated that PVL is associated with the number of infected cells, viral biokinetics, the BLV biodistribution in the organs during the early stages of experimentally infected cattle, viral infectivity, transmission potential and disease progression [15,16,17,18,19,20,21,22]. Further, previous studies have shown that the BLV provirus can be detected in the milk, nasal mucus, and saliva of dairy cattle with PVLs of >10,000, 14,000, and 18,000 copies/10^5^ cells in their blood samples, respectively [23,24]. Overall, these results suggest that a PVL of approximately 10,000 copies/10^5^ cells in blood may indicate efficient BLV spreading throughout the body, which is a relatively high number. Therefore, a BLV PVL of 10,000 copies/10^5^ cells was set as the threshold for distinguishing between high- and low-PVL cows [25,26]. Therefore, cattle maintaining a low PVL are reported to pose little detectable transmission risk to BLV-free herd mates, whereas a high or increasing PVL is associated with a greater risk of transmission [17,18,19,20,27,28,29]. Therefore, PVL provides a biologically and operationally meaningful phenotype for identifying the host factors influencing BLV control.

Host genetic variation is a major determinant of interindividual differences in PVL. The best characterized effect involves a highly polymorphic bovine leukocyte antigen (BoLA) region. *BoLA-DRB3* encodes the β-chain of a major histocompatibility complex (MHC) class II molecule that presents extracellularly derived peptides to CD4^+^ T cells; therefore, allelic variation at this locus is biologically relevant to antiviral immune recognition and presents a highly polymorphic gene located on bovine chromosome 23 (BTA23) [30]. In total, 387 alleles were registered in the immunopolymorphism database-major histocompatibility complex (IPD-MHC) database (https://www.ebi.ac.uk/ipd/mhc/group/BoLA/) (accessed on 27 July 2026). Among the alleles, in Japanese cattle populations, BoLA-DRB3*002:01, *009:02, and *014:01:01 have been used as resistance-associated alleles because their carriers tend to maintain lower BLV PVL or infectivity, whereas BoLA-DRB3*012:01 and *015:01 have been classified as susceptibility-associated alleles linked to higher PVL [19,25,26,28]. However, the effects of the *BoLA-DRB3* allele can vary with breed, population, heterozygosity, and epidemiological context [25,26,31,32,33,34,35,36,37,38,39,40,41]. Further, EBL is a multifactorial disease caused by a complex interplay between host genetic, environmental, and viral factors [1]. Therefore, because BLV disease susceptibility cannot be reliably determined based solely on polymorphism of the *BoLA-DRB3* gene, novel genomic markers related to BLV PVL in addition to *BoLA-DRB3* need to be identified.

Genome-wide association studies (GWAS) provide an unbiased approach for identifying additional host determinants. Previous GWAS have also identified several single-nucleotide polymorphisms (SNPs) related to BLV PVL in the BoLA region and elsewhere in the genome of Japanese Black and dairy cattle: one SNP within the spermatogenesis associated 16 (*SPATA16*)-coding region on BTA 1, which was evaluated across breeds [42], SNPs were located on BTA 23, 3, and 8 in close vicinity of patatin-like phospholipase domain containing 1 (*PNPLA1*), adaptor-related protein complex 4, beta 1 subunit (*AP4B1*), tripartite motif-containing 45 (*TRIM45*), and cell division cycle associated 2 (*CDCA2*) genes, respectively [43]; 1 SNP in the contactin 3 (*CNTN3)* gene on chromosome 22, and 2 in the BoLA region on BTA 23 [44], and 24 SNPs in close vicinity of genes related to immune response, cell cytoskeletal reorganization, the extracellular matrix genes and host transcription factors within the BoLA region [45]. However, several limitations have restricted the translation of these findings, including the use of medium-density arrays [43,44,45] and insufficient independent population-level validation [42,43,44,45]. Only a few association signals have been converted into rapid assays suitable for routine screening. Moreover, many GWAS variants are non-coding; therefore, association alone cannot identify the regulated gene or establish a causal mechanism.

The available control measures include hygiene management, segregation, selective replacement, and test-and-cull programs; however, their feasibility strongly depends on herd prevalence and economic circumstances [46]. Genetic markers that can reproducibly identify cattle that are predisposed to low PVL levels may complement PVL testing and herd management. Indeed, an integrated BLV eradication program using cattle with the resistant *BoLA-DRB3* allele as biological barriers and preferentially eliminating cattle with a susceptible *BoLA-DRB3* allele has shown practical potential [47]. A low-cost genotyping method would make it feasible to test candidate markers in larger herds and, following appropriate validation, incorporate them into marker-assisted or genomic selection. Such selection should complement rather than replace biosecurity and longitudinal infection monitoring. Kompetitive Allele-Specific PCR (KASP) is particularly attractive for this translational step because competitive allele-specific PCR enables the rapid and flexible genotyping of a small number of target variants across many samples and has been used for high-throughput MHC screening and marker-assisted selection [48,49,50].

In this study, we used a high-density Illumina Bovine HD BeadChip and a mixed-model GWAS implemented in GEMMA [51] to identify BLV PVL-associated SNPs in Holstein cattle. Furthermore, we developed KASP assays for the significant SNPs, and verified their genotype calls using Sanger sequencing. We also evaluated their association with PVL in an independent Holstein population. Finally, we evaluated the relationship between combined *BoLA-DRB3* and SNP marker status and low PVL; overall, the study was designed to bridge SNP discovery, independent validation, and assay development, with the long-term objective of developing scalable genetic tools for BLV control.

## 2. Materials and Methods

### 2.1. Study Populations and Sample Selection

Blood samples were collected for GWAS from 113 BLV-infected clinically asymptomatic Holstein cattle from BLV-positive farms and slaughter-inspection facilities in Japan. For independent validation using the KASP assay, blood samples from 364 Holstein cattle that were not included in the GWAS discovery population were used for targeted SNP genotyping and PVL association analysis. All animals were handled by veterinarians in accordance with the guidelines of the University of Tokyo. This study was approved by the Animal Experiments Committee of the University of Tokyo (Approval Number p22–2–030; Approval date, 22 May 2022).

### 2.2. Genomic DNA Extraction and BLV Proviral Load Quantification

Genomic DNA was extracted from 300 µL of ethylenediaminetetraacetic acid (EDTA)-treated whole blood using the Wizard Genomic DNA Purification Kit (Promega Corporation, Madison, WI, USA) according to the manufacturer’s protocol. DNA concentration and quality were evaluated using a NanoDrop Spectrophotometer ND-1000 (Thermo Fisher Scientific, Waltham, MA, USA). DNA was diluted to 30 ng/µL with Nuclease-Free Water (Thermo Fisher Scientific). The BLV-positive status was determined using quantitative PCR (qPCR).

PVL was quantified from genomic DNA by BLV-CoCoMo-qPCR using the THUNDERBIRD Probe qPCR Mix (Toyobo, Tokyo, Japan) and BLV-CoCoMo-qPCR reagents (Nippon Gene, Toyama, Japan), as described previously [13]. Amplification was performed using a LightCycler 480 System II (Roche Diagnostics, Mannheim, Germany). Finally, the PVL was calculated using the following formula: (number of BLV long terminal repeat (LTR) copies/number of *BoLA-DRA* copies) × 10^5^ cells. The BLV PVL was expressed as the number of copies per 10^5^ cells.

### 2.3. High-Density SNP Genotyping and Quality Control

In total, 113 samples were genotyped using an Illumina Bovine HD Genotyping BeadChip (Illumina, Inc., San Diego, CA, USA) targeting 770,962 SNPs. Quality control was performed using PLINK v1.9 [52,53]. SNPs were retained when the minor allele frequency was >0.01, the SNP call rate was >0.95, and the Hardy–Weinberg equilibrium test yielded *p* > 0.001. Samples with a call rate ≤ 0.95 were excluded. After filtering, 582,324 SNPs were retained.

### 2.4. Genome-Wide Association and Linkage Disequilibrium Analyses

Cattle were categorized as low PVL (0–999 copies/10^5^ cells; *n* = 37), intermediate PVL (1000–9999 copies/10^5^ cells; *n* = 43), or high PVL (10,000–120,482 copies/10^5^ cells; *n* = 33). For the primary extreme-phenotype analysis, high- and low-PVL groups were coded as a binary phenotype, and the intermediate group was excluded, yielding 70 animals for the primary association analysis. Genome-wide association testing was performed with GEMMA using a linear mixed model and genomic relatedness matrix [51]. Allelic odds ratios were calculated from allele counts in the high- and low-PVL groups to provide descriptive effect-size estimates.

The genome-wide significance threshold was set at *p* = 8.59 × 10^−8^ using Bonferroni correction (0.05/582,324) [54]. The genomic inflation factor (λGC) was calculated as the ratio of the median observed association test statistic to its expected median under the null hypothesis [55]. Association results were visualized as Manhattan plots using the qqman package in R [56]. Local linkage disequilibrium (LD) surrounding the two significant loci was evaluated with Haploview v4.2 by calculating r^2^ and D′ [57]. Variant positions and neighboring genes were annotated against the ARS-UCD1.2 bovine genome assembly.

### 2.5. KASP Genotyping

KASP assays targeting rs110742604 (SNP_K_10) and rs137368875 (SNP_K_11) were designed and synthesized by LGC Biosearch Technologies (Hoddesdon, UK). A total of 364 samples from BLV-infected cattle that were not included in the GWAS were used for the assay. Each 10 µL reaction contained 5 µL of 2× KASP-TF master mix, 0.14 µL of KASP assay mix, 4 µL of Nuclease-Free Water, and 1 µL of genomic DNA (30 ng/µL). Reactions were performed using the LightCycler 480 System II (Roche Diagnostics). The cycling program was 94 °C for 15 min; 10 touchdown cycles of 94 °C for 20 s and 61–55 °C for 60 s (decreasing by 0.6 °C per cycle); 26 cycles of 94 °C for 20 s and 55 °C for 60 s; and 30 °C for 60 s. Genotypes were assigned from allele-specific fluorescence clusters. The general suitability of KASP for targeted MHC screening and marker-assisted selection has been previously demonstrated [48,49,50].

### 2.6. Confirmation of KASP Genotypes Using Sanger Sequencing

To evaluate the KASP call accuracy, the genomic regions containing SNPk10 and SNPk11 were amplified from 61 and 22 samples, respectively, selected from the 364 cattle used in the validation study using PrimeSTAR GXL DNA Polymerase (TaKaRa Bio, Kusatsu, Japan). SNP_K_10 was amplified using forward primer 5′-AGATCCAGCAGAATCAAGAGAAG-3′ and reverse primer 5′-CACTCAAGAAGCCCAAGAGAA-3′ (475 bp). SNP_K_11 was amplified using forward primer 5′-CTCACTGAGGCTTGAAGAAGAA-3′ and reverse primer 5′-GGAGGACACCCAGCAATAAC-3′ (422 bp). The thermal cycle program comprised 98 °C for 10 s, followed by 30 cycles of 98 °C for 10 s, 60 °C for 15 s, and 68 °C for 1 min, with a final hold at 4 °C. Amplicons were purified using the FastGene Gel/PCR Extraction Kit (Nippon Genetics, Tokyo, Japan) and sequenced using Fasmac (Atsugi, Japan). Chromatograms were analyzed using SnapGene v8.0.1, and Sanger-derived genotypes were directly compared with KASP calls.

### 2.7. BoLA-DRB3 Genotyping

*BoLA-DRB3* alleles were determined using the PCR-sequence-based typing (SBT) method described by Takeshima et al. (2011) [58]. Briefly, *BoLA-DRB3* exon 2 was amplified through single-step PCR using the DRB3 forward (5′-CGCTCCTGTGAYCAGATCTATCC-3′) and reverse (5′-CACCCCCGCGCTCACC-3′) primer sets. The PCR products were purified using a FastGene Gel/PCR Extraction Kit (NIPPON Genetics Co., Ltd., Tokyo, Japan) and sequenced using FASMAC (Atsugi, Japan). Sequence data were analyzed using the Assign 400ATF ver. 1.0.2.45 software (Gonexio Genomics, Fremantle, Australia) to determine the *BoLA-DRB3* genotype. When one or two alleles were detected, the samples were considered homozygous or heterozygous.

### 2.8. Classification and Exploratory Analysis for Combined-Resistance Markers

The *BoLA-DRB3* genotypes available for the independent validation population were classified using previously applied resistance categories in Japanese Holstein cattle [25,28]: Cattle were divided into resistant, susceptible, and neutral groups based on the presence of *BoLA-DRB3* alleles, as follows: susceptible cattle carried at least one BoLA-DRB3*012:01 or *015:01 allele in their genomes; resistant cattle carried at least one BoLA-DRB3*002:01, *009:02, or *014:01:01 allele in their genomes; and neutral cattle carried other alleles in their genomes. Importantly, resistance is a dominant trait and susceptibility is a recessive trait [31]. Therefore, cattle carrying both susceptible and resistant alleles were defined as resistant. In this study, cattle carrying at least one BoLA-DRB3*002:01, *009:02, or *014:01:01 allele were classified as BoLA resistant (BoLA-R). Animals carrying both resistance- and susceptibility-associated alleles remained in the BoLA-R group. The three evaluated candidate resistance markers were the BoLA-R status, rs110742604 CC genotype (SNP_K_10-CC), and rs137368875 CC genotype (SNP_K_11-CC).

The exact overlap among the three markers was enumerated. Cattle were then grouped according to the number of candidate resistance markers carried (zero to three), and the PVL distributions and proportions of low-PVL (<1000 copies/10^5^ cells), intermediate-PVL (1000–9999 copies/10^5^ cells), and high-PVL (≥10,000 copies/10^5^ cells) animals were summarized. Two cattle lacking SNP_K_11 genotype calls were excluded, leaving 362 complete cases. PVL distributions across the marker-count groups were compared using the Kruskal–Wallis test. This analysis was pre-specified as exploratory and descriptive; it was not adjusted for farm or population covariates and was not used to infer independent, additive, or synergistic marker effects.

### 2.9. Statistical Analysis

PVL distributions among the TT, TC, and CC genotypes were compared using the Kruskal–Wallis test followed by Dunn’s multiple-comparison test with Bonferroni-adjust.

## 3. Results

### 3.1. Identification of Two PVL-Associated SNPs Using Genome-Wide Association Analysis

PVL is an important diagnostic index for determining disease progression and transmission risk in BLV infections. PVL in peripheral blood strongly correlates with infection status, transmission potential, and disease development [15,17,18,19,20,21]. Therefore, we used it to identify SNPs associated with BLV PVL in Holstein cattle using the high-density Illumina BovineHD BeadChip and a mixed-model GWAS implemented in GEMMA. First, PVL was measured in 113 dairy cattle using the BLV-CoCoMo-qPCR assay (Table 1). PVL values ranged from 0 to 120,482 copies per 10^5^ cells. Referring to previous studies [34,35,59], we classified cattle into three groups based on distribution: the top 30% as high PVL, the bottom 30% as low PVL, and the remaining animals as moderate PVL. In total, 113 BLV-positive Holstein cattle were classified according to PVL as low-PVL (0–999 copies per 10^5^ cells; *n* = 37), intermediate-PVL (1000–9999 copies per 10^5^ cells; *n* = 43), or high-PVL (10,000–120,482 copies per 10^5^ cells; *n* = 33). Next, we performed a GWAS using these traits as binary variables, as was done in 33 cases (high-PVL group) and 37 controls (low-PVL group). The 43 cattle with intermediate PVL were excluded from the extreme phenotypic comparison. The 70 animals were genotyped using the Illumina Bovine HD BeadChip targeting 770,962 SNPs. After quality control, 582,324 SNPs met the quality control criteria (call rate > 95%, minor allele frequency > 0.01, SNP call rate > 0.95, Hardy–Weinberg equilibrium, *p* > 0.001). Analyses were then performed using GEMMA, which uses a genetic relatedness matrix within a linear mixed-model framework to reduce confounding from the population structure [51].

The genomic inflation factor (λGC) for this analysis was 1.030164, indicating that the sample was appropriate for inclusion in the association study (Figure 1A). The quantile–quantile (Q–Q) plot showed that the two SNPs deviated significantly from the null hypothesis (Figure 1A). Genome-wide mixed-model analysis identified two SNPs that exceeded the Bonferroni-corrected significance threshold (*p* = 8.6 × 10^−8^; Figure 1B). SNP_K_10 (ARS-BFGL-NGS-4240; rs110742604) was located at 28,284,087 bp on BTA 23 and was associated with PVL at *p* = 1.18 × 10^−8^ (minor allele C; minor allele frequency = 0.199; odds ratio = 8.714; Table 2). SNP_K_11 (BovineHD2300008097; rs137368875) was located at 28,847,653 bp on BTA 23 and showed an association at *p* = 3.81 × 10^−8^ (minor allele C; minor allele frequency = 0.243; odds ratio = 8.429; Table 2).

### 3.2. Linkage Disequilibrium Analysis of SNP_K_10 and SNP_K_11

The two SNPs were mapped to the BoLA class I region on BTA23 and were separated by a 563,566 bp window located at 28,284,087–28,847,653 bp according to their reported ARS-UCD1.2 coordinates (Table 2). SNP_K_10 was situated 825 bp upstream of *IER3* and 97,226 bp downstream of *DDR1* genes, whereas SNP_K_11 was flanked by Tripartite Motif Containing 40 *(TRIM40)* and Tripartite Motif Containing 31 (*TRIM31)*. Approximately 35 annotated genes were located between these two loci. LD analysis did not place the two SNPs within a shared haplotype block in the analyzed population (Figure 1C). However, the SNP pair showed a strong allelic correlation (*r*^2^ = 0.78).

### 3.3. Development and Validation of KASP Genotyping Assays for SNP_K_10 and SNP_K_11

To follow up on the results of the GWAS, allele-specific KASP assays were developed for SNP_K_10 and SNP_K_11. Genotypes assigned using the optimized KASP assay were compared with those determined independently using conventional Sanger sequencing (Figure 2). Clear clustering of the CC, TC, and TT genotypes was observed for both assays. For SNP_k_10, KASP calls were concordant with Sanger sequencing in all 61 samples examined (61/61, 100%; Figure 2A,B). For SNP_k_11, the 22-sample nested subset likewise showed complete concordance (22/22, 100%; Figure 2C,D).

### 3.4. Validation of the Association Between SNP Genotypes and BLV PVL in an Independent Cattle Population for SNP_K_10 and SNP_K_11

To further confirm the GWAS results, the association between SNP genotypes for SNP_K_10 and SNP_K_11 and BLV PVL was investigated in 364 additional field samples collected in a nationwide survey across Japan using KASP genotyping assays. In addition, the 70 animals included in the GWAS were investigated using KASP genotyping assays for SNP_K_10 and SNP_K_11 (Data S2). SNP_K_10 was successfully typed in all animals, whereas SNP_K_11 was evaluated in 362 cattle except two animals. For the genotypes of SNP_K_10 with 364 samples, the sample sizes and mean PVL values were as follows: TT (*n* = 247, mean = 23,910 copies per 10^5^ cells), CC (*n* = 15, mean = 218 copies per 10^5^ cells), and TC (*n* = 102, mean = 11,967 copies per 10^5^ cells) (Figure 3A). PVL differed significantly among the TT, TC, and CC genotypes (Kruskal–Wallis test, *p* < 0.001). At SNP_K_10, animals with the TT genotype had a higher PVL than that of animals with either the TC or CC genotype (Dunn’s post hoc test, both *p* < 0.001), and the PVL also differed between the TC and CC animals (*p* < 0.01). A similar effect was observed for SNP_K_11. For the SNP_K_11 genotypes in 362 blood samples, the sample sizes and mean PVL values were as follows: TT (*n* = 231, mean = 25,108 copies per 10^5^ cells), CC (*n* = 24, mean = 3196 copies per 10^5^ cells), and TC (*n* = 107, mean = 11,579 copies per 10^5^ cells) (Figure 3B). The TT animals had a higher PVL than that of the TC or CC animals (both *p* < 0.001), whereas the TC and CC animals differed at *p* < 0.05. Thus, carriage of the C allele at either locus was consistently associated with reduced PVL in the validation population.

### 3.5. Combined-Marker Stratification by BoLA-DRB3 and SNP Genotypes

We then clarified an association between the combined markers *BoLA-DRB3*, SNP_K_10 and SNP_K_11, genotypes, and BLV PVL. The distribution of *BoLA-DRB3* alleles in 362 Holstein samples was first determined using PCR-SBT targeting exon 2 of the *BoLA-DRB3* gene and classified using the following resistance categories: cattle carrying at least one BoLA-DRB3*002:01, *009:02, or *014:01:01 allele (BoLA-R) were classified as resistant, in conjunction with the rs110742604 CC genotype (SNP_K_10-CC) and the rs137368875 CC genotype (SNP_K_11-CC). As shown in Figure 4A, among the 362 cattle with three complete markers, BoLA-R, SNP_K_10-CC, and SNP_K_11-CC, 53 carried BoLA-R, 15 had the SNP_K_10-CC genotype, and 24 had the SNP_K_11-CC genotype. The markers overlapped substantially: 12 cattle carried all three markers, 14 of the 15 SNP_K_10-CC cattle also harbored SNP_K_11-CC, and 12 of the 15 SNP_K_10-CC cattle were resistant. The exact intersections and their mean PVL values were as follows: 35 cattle with BoLA-R alone (8298 copies/10^5^ cells), no cattle with SNP_k_10-CC alone, five with SNP_k_11-CC alone (6423 copies/10^5^ cells), one with BoLA-R plus SNP_k_10-CC (93 copies/10^5^ cells), five with BoLA-R plus SNP_k_11-CC (8282 copies/10^5^ cells), two with SNP_k_10-CC plus SNP_k_11-CC (46 copies/10^5^ cells), 12 with all three markers (257 copies/10^5^ cells), and 302 without any of the three markers (22,346 copies/10^5^ cells). Among the seven cattle carrying SNP_k_10-CC and/or SNP_k_11-CC without BoLA-R, two had low PVL (46 copies/10^5^ cells) and five had intermediate PVL (6423 copies/10^5^ cells); none had high PVL. Across all 15 SNP_k_10-CC cattle, irrespective of BoLA-R status, the mean PVL was 218 copies/10^5^ cells; 14 had low PVL and none had high PVL.

Next, the cattle were divided into four groups based on the number of resistance markers (0 = cattle without marker, 1 = cattle carrying one marker, 2 = cattle carrying two markers, and 3 = cattle carrying three markers), and the distributions and proportions of PVL in each group were analyzed (Figure 4B,C). PVL decreased with an increase in the number of candidate resistance markers (Kruskal–Wallis, *p* < 0.001; Figure 4B). Median PVLs were 13,228 copies/10^5^ cells in cattle carrying no marker (*n* = 302), 350 copies/10^5^ cells in cattle carrying one marker (*n* = 40), 105 copies/10^5^ cells in cattle carrying two markers (*n* = 8), and 54 copies/10^5^ cells in cattle carrying all three markers (*n* = 12). The corresponding proportions of cattle with low PVL were 20.9%, 57.5%, 75.0%, and 91.7%, whereas the proportions of cattle with high PVL were 53.3%, 25.0%, 12.5%, and 0% in groups 0, 1, 2, and 3, respectively (Figure 4C). Eleven of the twelve cattle carrying all three markers were classified as having low PVL, and none were classified as having high PVL, indicating a consistent trend toward lower PVL as the number of candidate resistance markers increased. Although the marker-defined subgroups were small and overlapping, these findings support further evaluation of SNP_k_10 and SNP_k_11 as complementary markers to *BoLA-DRB3*.

## 4. Discussion

In this study, we explored BLV PVL-associated SNPs in Japanese Holstein cattle using the high-density Illumina BovineHD BeadChip and a mixed-model GWAS implemented in GEMMA. First, we successfully identified two PVL-associated variants, rs110742604 and rs137368875, in the BoLA class I region of BTA23 and reproduced their genotype–phenotype relationships in an additional Holstein population. SNP_K_10 (rs110742604) was previously reported by Carignano et al., 2018 [45], whereas SNP_K_11 (rs137368875) was discovered for the first time in this study. Second, the two SNPs exceeding the genome-wide significance threshold were separated by more than 0.5 Mb and were not contained within a common strong LD block but showed a strong allelic correlation (r^2^ = 0.78). Generally, clustering of genes with related functions in the LD pattern of the BoLA region makes it difficult to detect the effects of individual loci [60]. The strong allelic correlation and complexity of the BoLA region indicate that the relationships among the two SNPs and *BoLA-DRB3* should be further evaluated in larger datasets. Third, we demonstrated the effectiveness of the KASP system for the two SNPs. In the validation population, the C allele at either locus was associated with a lower PVL, and the lowest values were observed in the CC animals. In particular, exploratory integration with established *BoLA-DRB3* resistance-associated alleles further showed stepwise enrichment in low-PVL cattle as the number of candidate resistance markers increased; further, 11 of the 12 cattle carrying all three markers had low PVL, and none had high PVL. These genetic results were complemented by a rapid KASP workflow, which showed complete concordance with the Sanger sequencing results. These results support the two SNPs as candidate PVL-associated markers and the KASP assays as a practical platform for larger-scale validation and future evaluation in breeding and BLV control strategies.

Previous GWAS have identified several SNPs related to BLV PVL in the BoLA region and additional loci distributed across the bovine genome in Japanese Black and dairy cattle [25,26,31,32,42,43,44,45]. Our findings extend this genetic architecture by identifying two validated signals in the BoLA class I region. They reside within the broader BoLA region; however, they are distinct from the extensively studied *BoLA-DRB3* and *DQA1* class II markers and appear to represent separate association signals from one another, keeping a distance of more than 0.5 Mb, but they are related to only partially competing traits. Therefore, their strongest immediate value is as candidate biomarkers for risk stratification, not as proven causal variants.

However, the mechanisms controlling BLV PVL have not been fully elucidated. SNP_K_10 is located upstream of *IER3* and downstream of *DDR1*. *IER3* is an immediate-early response gene that integrates stress, inflammation, survival, and apoptotic signals [61,62]. *IER3* has also been implicated in the aggressive progression and autophagy occurring in acute myeloid leukemia through AKT/mTOR-related signaling [63]. These functions may be relevant to BLV, a deltaretrovirus that persists in long-lived lymphocytes and, in a minority of infected cattle, drives clonal expansion and lymphoma. However, direct evidence linking *IER3* to BLV replication and transformation remains lacking. In contrast, *DDR1* encodes a receptor tyrosine kinase activated by fibrillar collagen and transduces signals from the extracellular matrix into cells [64]. Pathogenic or disease-associated findings of *DDR1* have been reported in adenocarcinoma, melanoma, glioma susceptibility 1, and soft tissue sarcoma. However, an association between *IER3* and BLV or other retroviral replication has not been reported. Therefore, further research on the interaction between *IER3* or *DDR1* and BLV proviral integration and its impact on the regulation of BLV PVL remains indispensable. Another finding of our study is that SNP_K_11 lies between *TRIM40* and *TRIM31*. TRIM proteins commonly function as ubiquitin or ubiquitin-like modification regulators, and many TRIM family members participate in innate immune and antiviral pathways. *TRIM40* has also been shown to promote neddylation of IKKγ and to suppress NF-κB activity [65], providing a mechanistic link to inflammatory signaling that is potentially relevant to persistent retroviral infection. Nevertheless, *TRIM40* should not be labeled as an established BLV restriction factor because no direct evidence currently shows that it controls BLV entry, proviral transcription, clonal expansion, or virion production. *TRIM31* also regulates innate inflammation by promoting the proteasomal degradation of NOD-Like Receptor family Pyrin domain-containing 3 (NLRP3) [66], but this function has not been demonstrated in BLV infection. Therefore, further studies are needed to clarify the effects of BLV proviral regulation on *TRIM40* and *TRIM31* near SNP_K_11 reported in this study.

SNP_K_10 is located in the intergenic region between *IER3* and *DDR1*, whereas SNP_K_11 is located in the intergenic region between *TRIM40* and *TRIM31*. A large proportion of trait-associated variants identified using GWAS map to noncoding regulatory DNA, particularly cell type-specific open chromatin and enhancer elements [67]. Intergenic variants can alter transcription factor binding, enhancer activity, chromatin accessibility, or long-range enhancer–promoter contacts, and their target genes need not be the nearest annotated gene [68]. Consequently, *IER3*, *DDR1*, *TRIM40*, and *TRIM31* are reasonable candidates, and it is important not to conclusively identify them as effector genes but rather to simultaneously search for the true associated genes across a broader range.

Genetic association is useful for disease control only if it can be measured accurately and economically. Sanger sequencing is suitable for confirmation but requires post-PCR processing, sequence interpretation, and a greater per-sample time. In contrast, KASP uses endpoint allele-specific fluorescence and is well-suited for testing a small number of variants across many animals. KASP-derived markers have been used for high-throughput haplotype screening and marker-assisted selection in breeding programs [48,49,50]. In this study, both assays produced genotypes concordant with those of Sanger sequencing and generated results in approximately 90 min, supporting their use as practical candidate tests. Notably, clinical and breeding implementations require more than locus-specific analytical concordance (61 samples for SNP_k_10 and a nested subset of 22 samples for SNP_k_11) in this study. Therefore, assay performance, including call rate, repeatability, Mendelian consistency, sensitivity to DNA quality, and robustness to polymorphisms within primer-binding sites, should be quantified across laboratories and breeds. The two failed SNP_K_11 calls among the 364 attempted samples illustrate this need in the highly polymorphic BoLA region. Most importantly, prospective studies must establish predictive performance—effect size, discrimination, calibration, and incremental value beyond PVL measurement and established BoLA markers—before assays can be described as diagnostic tests.

*BoLA-DRB3* is one of the most reproducible genetic determinants of BLV PVL and infectivity. Its highly polymorphic peptide-binding region can alter the repertoire of viral peptides presented to CD4^+^ T cells, thereby contributing to differences in immune control [25,30]. In this study, the identification of two additional class I-region SNPs was compatible with PVL as a complex polygenic trait influenced by antigen presentation, innate immune regulation, viral factors, age, infection history, and management. Further, EBL is a multifactorial disease caused by a complex interplay between host genetic, environmental, and viral factors [1]. The combined-marker analysis showed a clear reduction in PVL among cattle carrying the SNP_k_10-CC genotype. The 35 cattle carrying BoLA-R alone had a mean PVL of 8298 copies/10^5^ cells, lower than the mean of 22,346 copies/10^5^ cells in cattle without any of the three markers but higher than the mean of 218 copies/10^5^ cells across all 15 SNP_k_10-CC cattle, irrespective of BoLA-R status. Fourteen of the 15 SNP_k_10-CC cattle had low PVL and none had high PVL. Thus, despite the small number of SNP_k_10-CC cattle, its association with reduced PVL was descriptively more pronounced than that of BoLA-R alone in this cohort. Because most SNP_k_10-CC cattle also carried BoLA-R and SNP_k_11-CC, larger studies are needed to clarify the contribution of each marker.

This study has several limitations. The discovery cohort contained only 113 animals, limiting the power to detect smaller effects. The estimated effect sizes of the identified SNPs may have been overestimated. Further analyses with a larger number of samples are thus necessary. Discovery and validation were restricted to Holstein cattle. Therefore, transferability to Japanese Black, Jersey, Brown Swiss, crossbred, and geographically distinct populations needs to be examined. Thus, larger studies across independent populations are needed to refine effect estimates and assess generalizability. The combined-marker groups were unbalanced (*n* = 302, 40, 8, and 12 for zero to three markers) and partly overlapping. The independence of SNP_k_10 and SNP_k_11 remains to be established by conditional analysis. Larger datasets will be useful for evaluating marker independence, LD with individual *BoLA-DRB3* resistance-associated alleles, and the potential incremental value of combined genotyping. Because these loci lie in a structurally complex and highly polymorphic BoLA region, dense local sequencing is required to exclude untyped causal variants and assembly- or mapping-related artifacts. PVL was evaluated at a single or limited number of time points, and potential confounders, such as age, farm, infection duration, and viral genotype, require more complete modeling. In addition, further studies evaluating PVL in specific lymphocyte subsets may provide additional insight into their roles in BLV transmission and disease progression. We were unable to identify a suitable publicly accessible individual-level Holstein BovineHD dataset containing both loci; therefore, breed-level marker frequencies should be estimated in larger independent Holstein populations.

The next priority is to elucidate PVL-regulatory mechanisms through functional analysis of genes near SNPs, replication in multiple breeds and independent herds, longitudinal PVL measurement, targeted sequencing and statistical fine-mapping, genotype-aware bulk and single-cell RNA sequencing and assay for transposase-accessible chromatin (ATAC) profiling, expression quantitative trait locus (eQTL) and allele-specific expression analyses, reporter assays and electrophoretic mobility shift assay (EMSA) experiments, clustered regularly interspaced short palindromic repeats (CRISPR)-based perturbation of candidate bases and regulatory elements, and three-dimensional (3D) chromatin mapping. If these studies confirm robust, independent predictive effects, rs110742604 and rs137368875 could be incorporated into multi-marker selection tools aimed at lowering herd-level PVL and transmission risks. Their contribution to BLV control would then lie not in replacing the existing measures but in adding a scalable genetic layer to an integrated eradication strategy.

In conclusion, this study identified rs110742604 and rs137368875 as two PVL-associated loci in the BoLA class I region and reproduced their genotype–phenotype associations in an additional Holstein population. Overall, the variants represent separate locally associated SNPs from one another, and the carriage of the C allele at either locus was consistently associated with a lower PVL. Rapid KASP assays accurately reproduced the Sanger-derived genotypes in the analytical validation set, providing a practical platform for larger population studies. The combined-marker analysis identified a three-marker subgroup in which 11 of 12 cattle had low PVL and none had high PVL. SNP_k_10-CC also showed a more pronounced descriptive reduction in PVL than that for BoLA-R alone in this cohort. These findings suggest that SNP_k_10 and SNP_k_11 may provide complementary information to *BoLA-DRB3*; however, validation in larger independent populations is warranted before application to risk stratification, breeding, or BLV control programs. Practical application would also require consideration of economic feasibility, ethical aspects, and implementation in breeding programs.

## Figures and Tables

**Figure 1:**
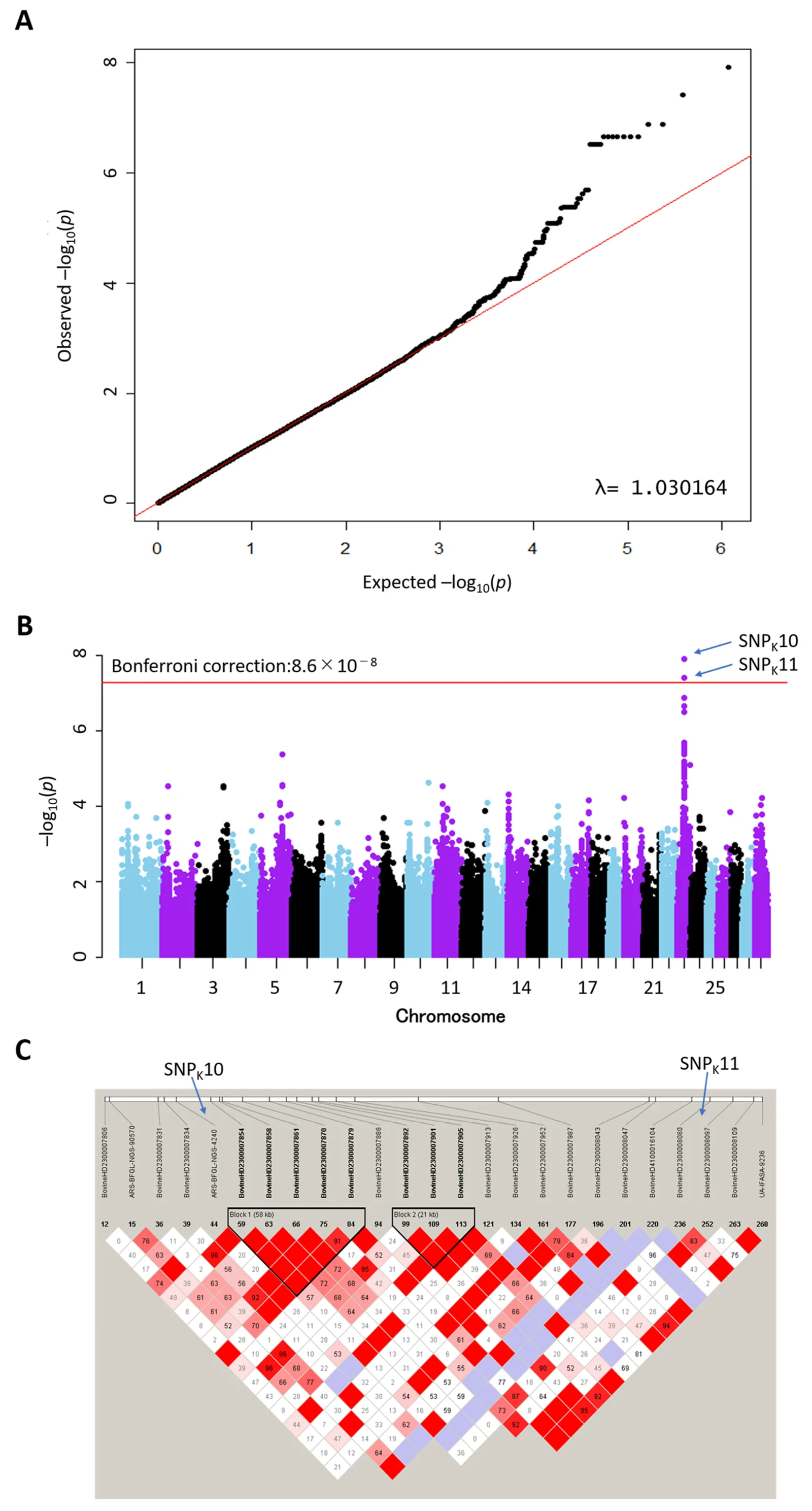
Genome-wide association and linkage-disequilibrium analyses of BLV proviral load. (**A**) Quantile–quantile (Q–Q) plot of the binary-phenotype genome-wide association analysis comparing 33 high-PVL and 37 low-PVL Holstein cattle using 582,324 quality-controlled SNPs. The genomic inflation factor (λ) is shown. (**B**) Manhattan plot of the same GWAS. The red horizontal line indicates the Bonferroni-corrected threshold (*p* = 8.6 × 10^−8^), and SNP_K_10 and SNP_K_11 on chromosome 23 exceeded this threshold. (**C**) Linkage-disequilibrium plot surrounding SNP_K_10 and SNP_K_11 in the BoLA class I region. The two loci were not contained within a common strong LD block.

**Figure 2:**
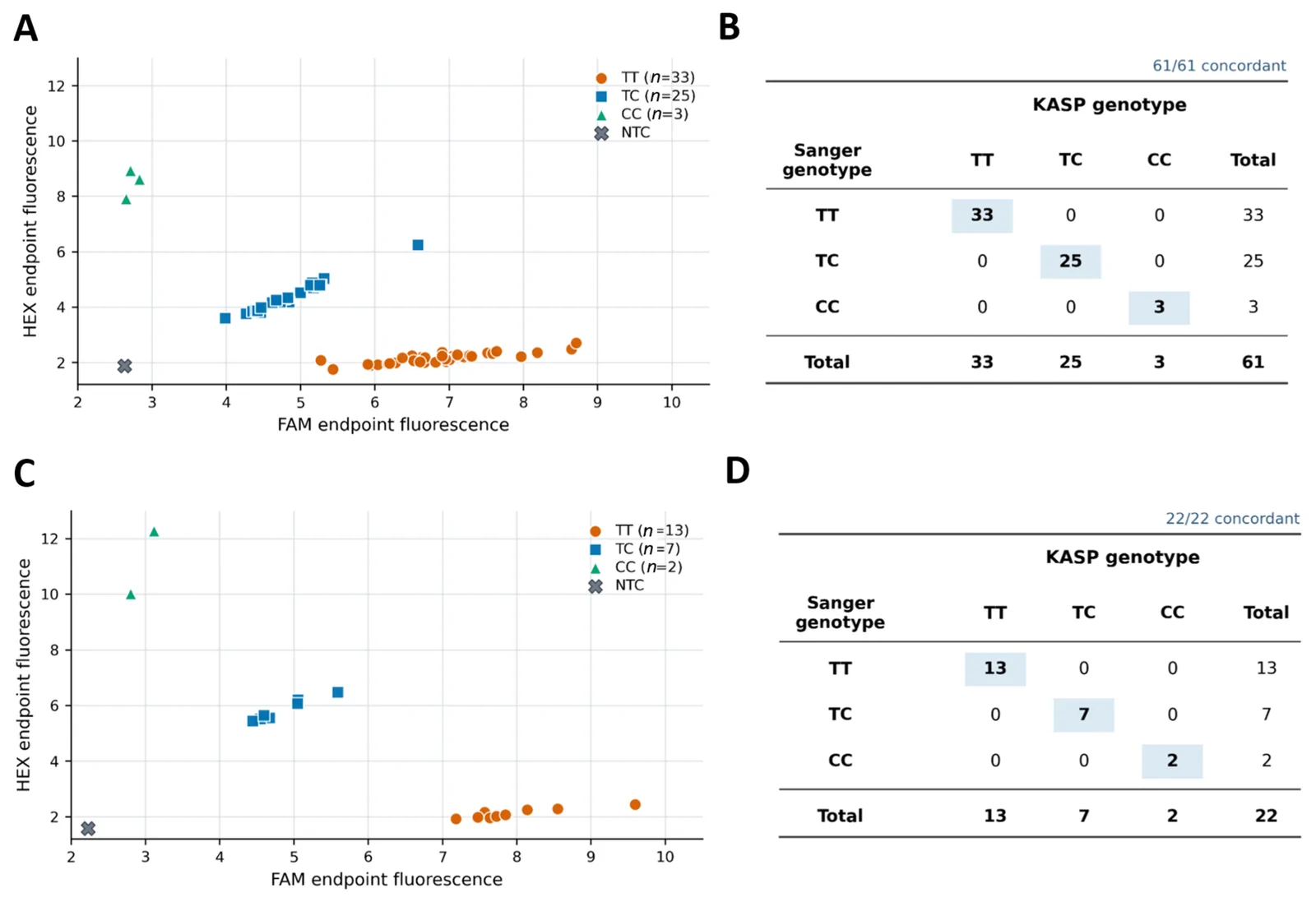
Development and analytical validation of KASP genotyping assays for the two PVL-associated SNPs. (**A**) Endpoint fluorescence cluster plot for rs110742604 (SNP_K_10). (**B**) Concordance table comparing KASP and Sanger sequencing genotypes for SNP_K_10. (**C**) Endpoint fluorescence cluster plot for rs137368875 (SNP_K_11). (**D**) Concordance table comparing KASP and Sanger sequencing genotypes for SNP_K_11. FAM and HEX endpoint fluorescence values were obtained from the KASP assays, and no-template controls (NTC) are shown in gray. The analytical validation included 61 samples for SNP_k_10 (33 TT, 25 TC, and 3 CC) and a nested subset of 22 of the 61 samples for SNP_k_11 (13 TT, 7 TC, and 2 CC); all were selected from the 362-cattle follow-up KASP cohort.

**Figure 3:**
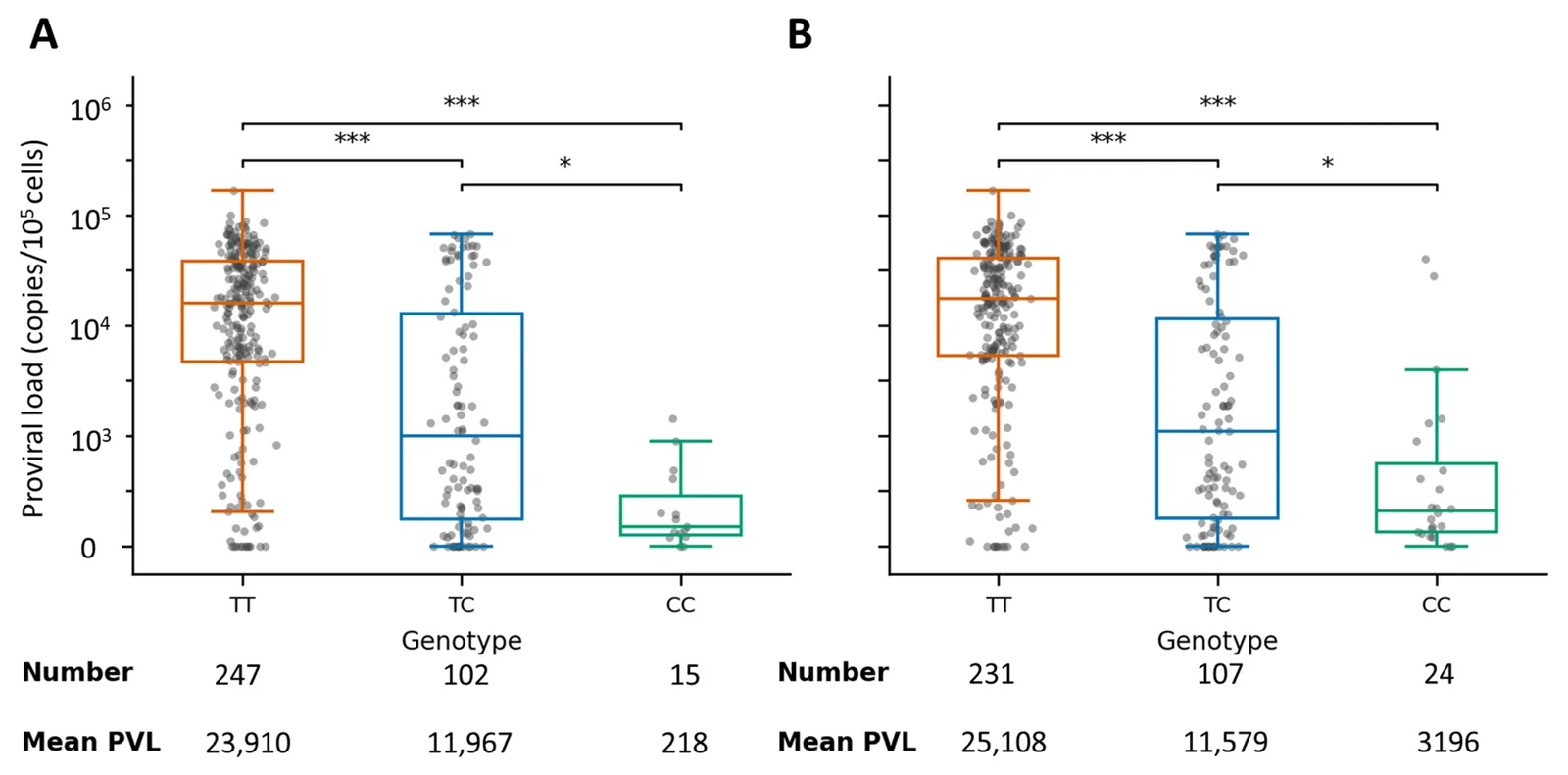
Association between SNP genotype and BLV proviral load in the independent validation population. (**A**) SNP_K_10 (*n* = 364) and (**B**) SNP_K_11 (*n* = 362). Points represent individual cattle, and boxes show the median and interquartile range. The boxplots and individual points display proviral load (copies/10^5^ cells), whereas the sample number and arithmetic mean PVL shown below each genotype are reported on the original scale (copies per 10^5^ cells). Group differences were assessed by the Kruskal–Wallis test followed by Dunn’s multiple-comparison test with Bonferroni-adjusted *p* values. * *p* < 0.05, *** *p* < 0.001. These PVL and KASP genotyping results are presented in Data S2.

**Figure 4:**
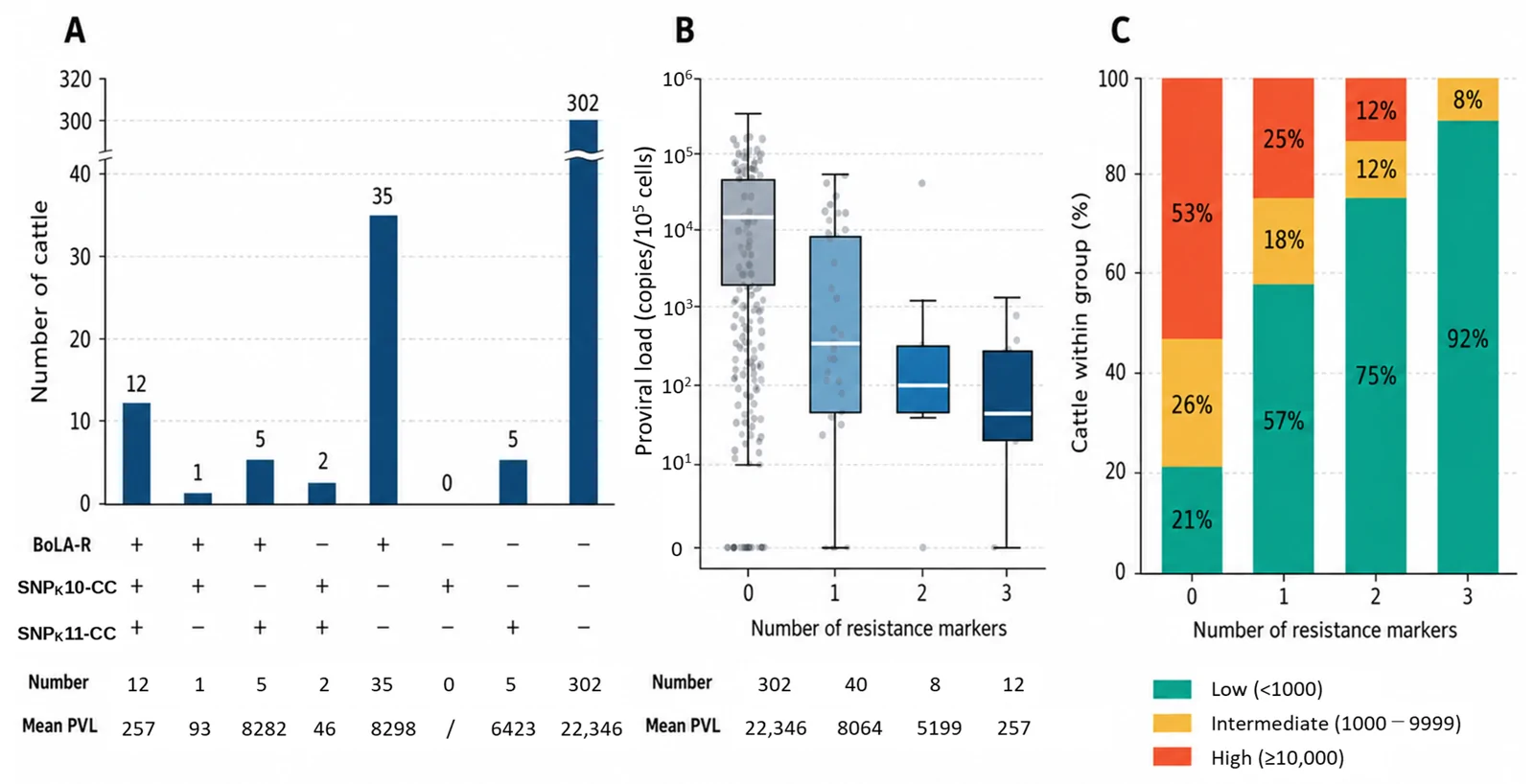
Exploratory combined stratification using *BoLA-DRB3* and the two PVL-associated SNPs. (**A**) Exact intersections among *BoLA-DRB3* resistance-associated status (BoLA-R), rs110742604-CC (SNP_K_10-CC), and rs137368875-CC (SNP_K_11-CC), including cattle carrying none of the three candidate resistance markers. Sample number and mean PVL for each exact intersection are shown below the plot; the bar for the no-marker group is truncated with a break because of its larger sample size. (**B**) Distribution of PVL according to the number of candidate resistance markers carried. Boxes indicate the median and interquartile range, points represent individual cattle, and the sample number and mean PVL for each marker-count group are shown below the x-axis. (**C**) Proportions of low-PVL (<1000 copies/10^5^ cells), intermediate-PVL (1000–9999 copies/10^5^ cells), and high-PVL (≥10,000 copies/10^5^ cells) cattle in each marker-count group. The analysis included 362 cattle with complete data for all three markers. These PVL and KASP genotyping results as well as *BoLA-DRB3* allele information are presented in Data S2.

**Table 1:** Classification of BLV-positive Holstein cattle according to proviral load. ^1^ BLV proviral load is expressed as copies per 10^5^ cells. The HPVL and LPVL groups (see Data S1) were used for the binary-phenotype GWAS.

PVL Group	PVL Range ^1^	Number of Cattle
Low PVL (LPVL)	0–999	37
Intermediate PVL	1000–9999	43
High PVL (HPVL)	10,000–120,482	33
Total	—	113

**Table 2:** Basic information for the two SNPs significantly associated with BLV proviral load. ^1^ Coordinates are based on the ARS-UCD1.2 bovine genome assembly. Both variants are located in intergenic regions. MAF, minor allele frequency.

SNP	Illumina ID	Chr.	rs ID	Position ^1^	Minor Allele	MAF	Major Allele	Odds Ratio	*p* Value	Nearest Genes
SNP_K_10	ARS-BFGL- NGS-4240	23	rs110742604	28,284,087	C	0.199	T	8.714	1.18 × 10^−8^	*IER3*/*DDR1*
SNP_K_11	BovineHD 2300008097	23	rs137368875	28,847,653	C	0.243	T	8.429	3.81 × 10^−8^	*TRIM40*/*TRIM31*

## Data Availability

The nucleotide sequences generated in this study have been deposited in the DDBJ/ENA/GenBank databases under accession numbers LC946423–LC946426. All other data generated or analyzed during this study are included in this article and its supplementary information files.

## Supplementary Materials

The following supporting information can be downloaded at https://www.mdpi.com/article/10.3390/pathogens15101017/s1: Data S1: GWAS discovery cohort. Data S2: validation cohort.
